# A retrospective multicenter study on clinical and serological parameters in patients with MuSK myasthenia gravis with and without general immunosuppression

**DOI:** 10.3389/fimmu.2024.1325171

**Published:** 2024-04-23

**Authors:** Inga Koneczny, Marina Mané-Damas, Shenghua Zong, Sander De Haas, Saif Huda, Daan van Kruining, Jan Damoiseaux, Anna De Rosa, Michelangelo Maestri, Melania Guida, Peter Molenaar, Philip Van Damme, Andreas Fichtenbaum, Thomas Perkmann, Marc De Baets, Konstantinos Lazaridis, Vasiliki Zouvelou, Socrates Tzartos, Roberta Ricciardi, Mario Losen, Pilar Martinez-Martinez

**Affiliations:** ^1^Research Group Neuroinflammation and Autoimmunity, Department of Psychiatry and Neuropsychology, School for Mental Health and Neuroscience, Maastricht University, Maastricht, Netherlands; ^2^Division of Neuropathology and Neurochemistry, Department of Neurology, Medical University of Vienna, Vienna, Austria; ^3^Neurosciences Group, Nuffield Department of Clinical Neurosciences, Weatherall Institute of Molecular Medicine, University of Oxford, Oxford, United Kingdom; ^4^Department of Neurology, Walton Centre National Health Service (NHS) Foundation Trust, Liverpool, United Kingdom; ^5^Department of Psychiatry and Neuropsychology, Maastricht University, Maastricht, Netherlands; ^6^Central Diagnostic Laboratory, Maastricht University Medical Center, Maastricht, Netherlands; ^7^Department of Clinical and Experimental Medicine, Neurology Unit, University of Pisa, Pisa, Italy; ^8^Neurology Department, University Hospital, Leuven, Belgium; ^9^Department of Neurosciences, Center for Brain & Disease Research, VIB, Leuven, Belgium; ^10^Department of Laboratory Medicine, Medical University of Vienna, Vienna, Austria; ^11^Department of Immunology, Hellenic Pasteur Institute, Athens, Greece; ^12^1^st^Neurology Department, National and Kapodistrian University of Athens, Athens, Greece; ^13^Department of Neuroimmunology, Tzartos NeuroDiagnostics, Athens, Greece; ^14^Cardio Thoracic and Vascular Surgery Department, University of Pisa, Pisa, Italy

**Keywords:** MuSK myasthenia gravis, IgG4, corticosteroids, prednisolone, prednisone, IgG4 autoimmune disease

## Abstract

**Introduction:**

Muscle-specific kinase (MuSK)- myasthenia gravis (MG) is caused by pathogenic autoantibodies against MuSK that correlate with disease severity and are predominantly of the IgG4 subclass. The first-line treatment for MuSK-MG is general immunosuppression with corticosteroids, but the effect of treatment on IgG4 and MuSK IgG4 levels has not been studied.

**Methods:**

We analyzed the clinical data and sera from 52 MuSK-MG patients (45 female, 7 male, median age 49 (range 17–79) years) from Italy, the Netherlands, Greece and Belgium, and 43 AChR-MG patients (22 female, 21 male, median age 63 (range 2–82) years) from Italy, receiving different types of immunosuppression, and sera from 46 age- and sex-matched non-disease controls (with no diagnosed diseases, 38 female, 8 male, median age 51.5 (range 20–68) years) from the Netherlands. We analyzed the disease severity (assessed by MGFA or QMG score), and measured concentrations of MuSK IgG4, MuSK IgG, total IgG4 and total IgG in the sera by ELISA, RIA and nephelometry.

**Results:**

We observed that MuSK-MG patients showed a robust clinical improvement and reduction of MuSK IgG after therapy, and that MuSK IgG4 concentrations, but not total IgG4 concentrations, correlated with clinical severity. MuSK IgG and MuSK IgG4 concentrations were reduced after immunosuppression in 4/5 individuals with before-after data, but data from non-linked patient samples showed no difference. Total serum IgG4 levels were within the normal range, with IgG4 levels above threshold (1.35g/L) in 1/52 MuSK-MG, 2/43 AChR-MG patients and 1/45 non-disease controls. MuSK-MG patients improved within the first four years after disease onset, but no further clinical improvement or reduction of MuSK IgG4 were observed four years later, and only 14/52 (26.92%) patients in total, of which 13 (93.3%) received general immunosuppression, reached clinical remission.

**Discussion:**

We conclude that MuSK-MG patients improve clinically with general immunosuppression but may require further treatment to reach remission. Longitudinal testing of individual patients may be clinically more useful than single measurements of MuSK IgG4. No significant differences in the serum IgG4 concentrations and IgG4/IgG ratio between AChR- and MuSK-MG patients were found during follow-up. Further studies with larger patient and control cohorts are necessary to validate the findings.

## 1 Introduction

Muscle-specific kinase (MuSK)-myasthenia gravis (MuSK-MG) is a severe autoimmune disease of the neuromuscular junction (NMJ) (1, 2). This condition is characterized by autoantibodies targeting MuSK, a pivotal tyrosine kinase crucial for NMJ development and maintenance (3). MuSK autoantibodies belong predominantly (approximately 90%) to the IgG4 subclass (4–8) that blocks the binding of MuSK to its direct binding partner, low-density lipoprotein receptor-related protein 4 (Lrp4), thereby interrupting a vital signal transduction pathway essential for the maintenance of the NMJ architecture (6, 9). MuSK-MG belongs to the IgG4 autoimmune diseases (IgG4-AIDs) (10, 11), which share common features such as low disease prevalence, predominance of IgG4 subclass antibodies with blocking as a pathogenic mechanism, and HLA associations (12–14).

The first-line treatment of patients with MuSK-MG consists of general immunosuppression with corticosteroids such as prednisone, often in combination with azathioprine. Even though it has been generally accepted to be a reliable treatment, a fraction of patients with MG remains treatment resistant and more difficult to manage clinically. Recent literature suggested general immunosuppression to be a less efficient treatment for MuSK-MG compared to AChR-MG (15, 16). B-cell depletion with rituximab has shown clinical benefit in MuSK-MG and other IgG4-AIDs (13, 17–21), and led to the reduction of MuSK IgG4 (22), but there is a lack of studies analyzing total IgG4 and MuSK IgG4 levels in patients with general immunosuppression.

In this study, we analyzed disease severity [assessed by Myasthenia Gravis Foundation of America (MGFA) or quantitative myasthenia gravis (QMG) score] in relation to MuSK IgG4, MuSK IgG, total IgG4, and total IgG concentrations in serum or plasma from patients with MuSK or AChR MG with and without immunosuppression and non-disease individuals, measured by enzyme-linked immunosorbent assay (ELISA), radioimmunoassay (RIA), and nephelometry.

## 2 Materials and methods

### 2.1 Patients

The study had a cross-sectional design, as the patient samples were taken at the same time as the clinical scoring. We also had additional follow-up serum of five patients from Greece available. All patient material was obtained with approval from the relevant ethical boards and after informed consent of the patients. We used serum or plasma from 52 patients with MuSK-MG: 45 female and 7 male patients; median age of onset, 43 years (range, 10–79 years); median age at sample, 49 years (**Table 1** and **Data Table 1**). Among these, 13 sera were from treatment-naïve patients with MuSK-MG (1 from the Netherlands, 1 from Belgium, 6 from Italy, and 5 from Greece) and 44 samples were from patients with MuSK-MG receiving general immunosuppression (36 from Italy, 2 from Belgium, 1 from the Netherlands, and 5 from Greece). The five patients from Greece had samples before and after long-term immunosuppression and were therefore included in both groups (for each of the five different patients, we included one sample in the before treatment group and one sample in the after treatment group; **Table 1** and **Data Tables 1** and **2**). The patients received immune therapy consisting of prednisone, azathioprine, intravenous immunoglobulins (IVIGs), cyclosporine, or combinations thereof (**Data Table 1**); these treatments are collectively referred to as “general immunosuppression”. This term was chosen to highlight the distinction from the more specific B-cell depletion therapy involving rituximab. At the time of sample collection, we included all patients with MuSK-MG, but rituximab was not established for the treatment of MuSK-MG at the clinic in Pisa. We observed that from other clinical centers (the Netherlands and Belgium), two patients receiving rituximab were in the cohort; this sample size was considered insufficient for a meaningful statistical analysis and were therefore excluded from the study. Five patients with MuSK-MG received IVIG in combination with other treatments (three in combination with prednisone and two in combination with cyclosporine and prednisone, all in intervals of 1–3 months; **Data Table 1**), which theoretically could affect serum IgG levels, but in all cases, blood sampling was prior to the IVIG infusion. The MGFA scores of the patients were between I and V, or they were in clinical remission. In tables and figures requiring numerical values, patients in clinical remission were represented as “0” to ensure their inclusion in the datasets. We defined a clinical improvement as a reduction of the MGFA score by a minimum of one point, and patients with unchanged or increased MGFA as not improved. The absence of AChR antibodies was confirmed by RIA. The clinical information is summarized in **Data Table 1**. Sera from 45 age- and sex-matched non-disease controls (Sanquin, Amsterdam) were used as controls: 38 female and 8 male controls; median age at sample, 51.5 years (range, 20–68 years). Furthermore, sera from 43 patients with AChR-MG (all from Italy), namely, 22 female and 21 male patients, with a median age of 63 years (range, 2–82 years) were used. Of these, 15 patients were untreated and 28 patients received general immunosuppression (summary in **Table 1** and details in **Data Table 1**). Clinical data were collected at the same time as blood samples during routine clinical checkups by the treating physician. The serum/plasma samples were analyzed using the tests outlined below. Owing to unavailable or low serum/plasma volumes or missing clinical data in a subset of samples, not all sera could be analyzed for every parameter.

**Table 1:** Summarized clinical information (details in **Supplementary Table 1**).

	MuSK-MG	AChR-MG	Non-disease controls
*Female, n* (%)	45 (86.54%)	22 (51.16%)	38 (82.61%)
*Male, n (%)*	7 (13.46%)	21 (48.84%)	8 (17.39%)
*Age at sample, median (range)*	49 (17–79)	63 (2–82)	47.8 (20–68)
*Age of onset, median (range)*	43 (10–79)	62 (1–82)	
*General immune therapy, n* (%)	39 (75%)/44 84.62%)*	28 (65.1%)	
*Oral corticosteroids (*prednisone, dexamethasone*)*	26 (50%)	19 (44.19%)	
*Prednisone and* a*zathioprine*	7 (13.46%)	6 (13.95%)	
*Prednisone and IVIG*	3 (5.77%)	0	
*Prednisone, plasmapheresis*	2 (3.85%)	0	
*Azathioprine only*	1 (1.92%)	0	
*Other combinations (see* **Supplementary Table 1***)*	5 (9.62%)	3 (6.98%)	
*Thymectomy*	5 (9.61%)	7 (16.28%)	

*Five patients with MuSK-MG from Greece had samples before and after treatment (details in **Table 2**).

### 2.2 MuSK IgG and MuSK IgG4 measurement

Total MuSK autoantibody levels were assessed by RIA (RSR, UK) according to the manufacturer’s instructions. MuSK IgG4 levels were measured by ELISA. To this end, ELISA plates (Microlon, catalog number 655092, Greiner, Austria) were coated with 1 µg/mL MuSK extracellular domain (ECD) produced in mammalian cells (a kind gift of Dr. Bernard Rees-Smith, RSR, UK). Bound MuSK antibodies were detected with mouse anti-human IgG4:HRP (1:3,500, catalog number MCA2098P, AbD Serotec, Germany). Samples were incubated with substrate containing 3,3′,5,5′-tetramethylbenzidine, and absorbance at 450 nm was measured using a VictorX3 plate reader (PerkinElmer, USA).

### 2.3 IgG quantification

IgG levels in patient sera were analyzed by ELISA, an immunoassay readily available in our research laboratory. ELISA plates (Microlon, catalog number 655092, Greiner, Austria) were coated with goat F(ab)_2_ anti-human IgG Fcγ (1:200, catalog number 109-006-008, Jackson ImmunoResearch, USA). Samples were added together with a standard dilution series of mAb-637 IgG1 (23). Bound antibodies were detected with goat F(ab)2 anti-human IgG Fcγ conjugated to HRP (1:20,000, catalog number 109-036-008, Jackson ImmunoResearch, USA). Samples were incubated with substrate containing 3,3′,5,5′-tetramethylbenzidine, and absorbance at 450 nm was measured using a VictorX3 plate reader (PerkinElmer, USA). IgG concentrations were verified using nephelometry.

### 2.4 IgG4 quantification

Human IgG4 serum/plasma concentrations were determined using particle-enhanced immune nephelometry with the BN II System (BN II Nephelometer, Siemens, Germany). Quantification of IgG4 levels by ELISA was not established. Hence, the methodology of the routine clinical laboratory was chosen after establishing that there was a good correlation between ELISA and nephelometry for total IgG (**Supplementary Figure 2A**).

### 2.5 Statistics

Statistical analyses were conducted using GraphPad Prism software version 9. Data were analyzed by normality and lognormality tests. In graphs with three or more datasets and Gaussian distribution, one-way analysis of variance (ANOVA) with Tukey post-hoc test was used. When data failed the normality test, nonparametric Kruskal**–**Wallis test and Dunn’s multiple comparisons test were used instead, and data were presented with median. In graphs with two datasets that showed a normal distribution, two-tailed *t*-test was used. Datasets without normal distribution and unmatched samples were analyzed using the Mann–Whitney test and datasets without normal distribution and paired data were analyzed with the Wilcoxon matched-pair signed**-**rank test. XY data were analyzed by linear regression followed by correlation analysis using the Pearson correlation efficient in case of normally distributed data, or the Spearman correlation coefficient in case the data did not show Gaussian distribution.

MuSK IgG4 and total IgG4 data were checked for normality, log-transformed, and analyzed using RStudio (version RStudio 2023.06.0 + 421). Linear regression models were constructed to assess the relationship (α = 0.05) between receiving immunosuppressive therapy and MuSK IgG4 levels. Additionally, age, sex, and MGFA scores were included as covariates. Furthermore, the association between immunosuppressive therapy and total IgG4 levels was investigated, and the same covariates were analyzed. Finally, the relationship between disease status (MuSK/AChR-MG) and total IgG4 levels was assessed, and the covariates age, sex, and immunosuppressive therapy were included in the model. An alpha level of 0.05 was used as the threshold for statistical significance.

## 3 Results

### 3.1 Immune therapy with clinical improvement was associated with reduced MuSK IgG levels

**Figure 1:**
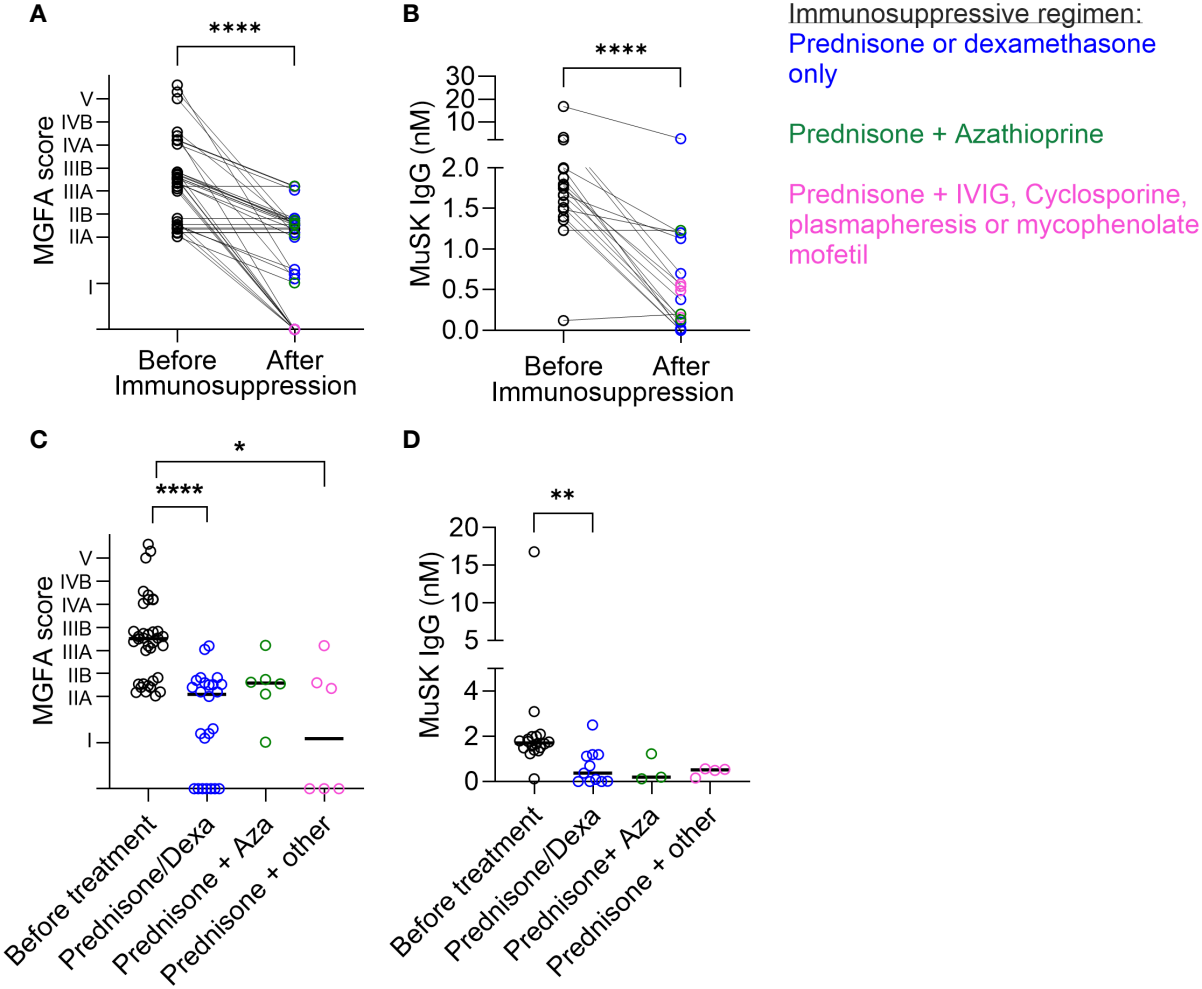
Disease severity and MuSK IgG levels were reduced after immunosuppression. **(A)** MGFA scores of patients with MuSK-MG at onset and at follow-up time after receiving immunosuppression. ****p < 0.0001, Wilcoxon matched-pair signed-rank test. To increase visibility, the lines of the MGFA scores were nudged slightly up or down. **(B)** MuSK IgG levels (determined at the time of diagnosis and the last follow-up) were reduced after receiving immunosuppressive treatment. Wilcoxon matched-pair signed-rank test, ****p < 0.0001. Comparison of **(C)** MGFA scores and **(D)** MuSK IgG titers (nM) before and after distinct treatments. Treatment is indicated as follows: blue = prednisone or dexamethasone, green = prednisone in combination with azathioprine, and pink = prednisone in combination with IVIG, cyclophosphamide, plasmapheresis, or mycophenolate mofetil. Kruskal–Wallis with Dunn’s post test. ****p < 0.0001, **p = 0.0013, *p = 0.0103. To include samples from patients with clinical remission in the graphs, these were given a value of “MGFA = 0”.

First, we assessed whether patients with MuSK-MG showed a clinical benefit from treatment with general immunosuppression. MGFA data from two time points (onset: before immunosuppression and follow-up: after immunosuppression) of 37 patients (36 Italy and 1 Belgium) receiving general immunosuppression were available. Following the diagnosis, where the patients were treatment naïve, the patients received immunosuppressive and immunomodulatory treatments, including oral corticosteroids alone (26 patients) or prednisone in combination with further treatments [azathioprine, IVIG, plasmapheresis, mycophenolate mofetil, or cyclosporine (11 patients)] (see **Data Table 1** for details). A significant reduction in MGFA scores after treatment was found (**Figure 1A**, *p* < 0.0001, Wilcoxon matched-pair signed**-**rank test). An absence of symptoms at follow-up was only observed in 10 out of 37 patients. When analyzing all patients with MuSK-MG, independent of their treatment, only 14/52 patients (26.9%) reached clinical remission (**Data Table 1** and **Table 1**). Additionally, MuSK IgG levels, as reported by the local diagnostic centers, demonstrated a decrease in patients undergoing immunosuppression (**Figure 1B**, *p* < 0.0001, Wilcoxon matched-pair signed**-**rank test) and MuSK IgG levels correlated with the MGFA scores of the patients (**Supplementary Figure 1**). When analyzing clinical severity after different immunosuppressive treatments, prednisone or dexamethasone alone (p < 0.0001) or in combination with other immunosuppressive treatment (p=0.0103) led to significant reductions in the MGFA score (**Figure 1C**). Treatment with prednisone or dexamethasone alone also led to a significant reduction of MuSK IgG levels (p= 0.0013, Kruskal Wallis test and Dunn's multiple comparison test, **Figure 1D**).

### 3.2 Clinical improvement was not significantly associated with reduced MuSK IgG4 or total IgG4 concentrations

Next, we analyzed whether MuSK IgG4, total IgG4, and total IgG levels were similarly reduced in patients with MuSK-MG. In the Italian/Belgian cohort, before–after immunomodulatory treatment sera/plasma were not available to assess these parameters. However, matched pairs of serum/plasma from individual patients before and after treatment were available from five patients from Greece (see summarized clinical information in **Table 2**).

**Table 2:** Clinical data of five Greek patients with MuSK-MG before and after immunosuppressive treatment.

Patient code	Sex	Age at diagnosis	Duration of treatment (months)	Treatment status	MuSK Ab concentration (nM, cutoff for positivity: >0.030)	Time treatment started after onset	QMG	Treatment (immunosuppression, IS)
*G1*	M	79	54 months	Before	27	0.6 months (20 days)	4	No IS
				After	21		0	Prednisone
*G2*	F	17	60 months	Before	11	2 months	2	No IS
				After	5.5		0	Prednisone
*G3*	F	45	6 months	Before	0.045	1 month	3	No IS
				After	0.1		1	Prednisone
*G4*	F	47	39 months	Before	n.a.	6 months	8	No IS
				After	1.8		4	Prednisone/Cyclosporine
*G5*	F	49	33 months	Before	13	3 months	9	No IS
				After	4.5		3	Prednisone/Mycophenolate mofetil

IS, immunosuppression.

**Figure 2:**
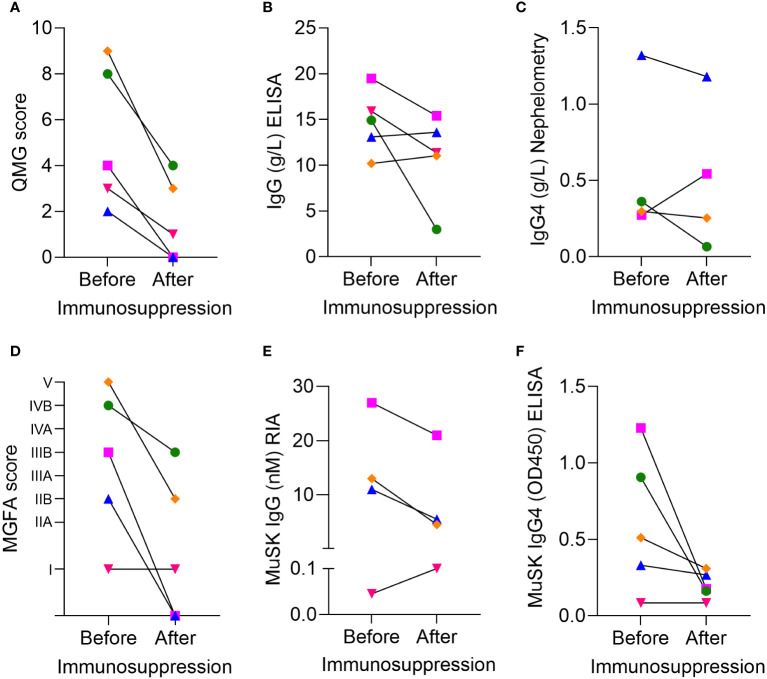
Clinical severity and serological data of five patients with MuSK-MG from Greece before and after immunosuppressive treatment. **(A)** Clinical severity by QMG score, **(B)** IgG concentrations measured by ELISA, **(C)** IgG4 concentrations measured by nephelometry, **(D)** MGFA scores. Pharmacological remission is indicated as 0. **(E)** MuSK IgG concentration assessed by RIA, **(F)** MuSK IgG4 levels measured by ELISA. Colors indicate individual patients: pink = G1, blue = G2, red = G3, green = G4, and orange =G5. Because of the limited serum volume, the IgG4 of patient G3 and the MuSK IgG of patient G4 could not be assessed. Statistical analysis was not considered appropriate due to the low sample number.

The before–after treatment samples indicated a clinical improvement in QMG scores (**Figure 2A**), which did not correlate with a consistent reduction in the total IgG levels (**Figure 2B**) or total IgG4 levels (**Figure 2C**), but with clinical change assessed by MGFA scores (**Figure 2D**), a reduction of MuSK IgG levels in three of four patients (**Figure 2E**) and MuSK IgG4 levels in four of five patients (**Figure 2F**).

However, unlike for MuSK IgG4, total serum IgG4 concentrations (**Figure 2C**) and total IgG levels were not consistently reduced after treatment. Due to the low number of patients, we assessed whether these results could also be reproduced in the larger patient cohort with additional samples from non-matched patients with and without immunosuppression from Italy, the Netherlands, and Belgium.

**Figure 3:**
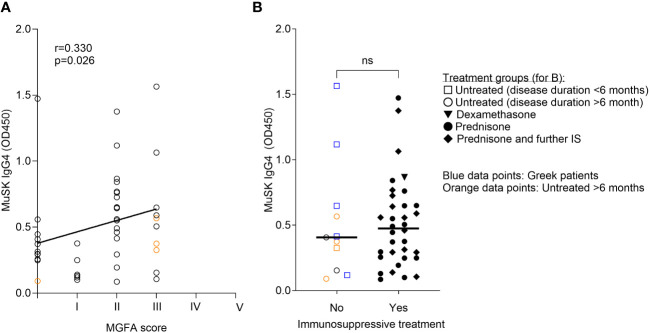
MuSK IgG4 levels correlated with clinical severity, but not with treatment status. MuSK IgG4 concentrations were measured by ELISA. **(A)** MuSK IgG4 concentrations (OD450) correlated with clinical severity (MGFA score). Linear regression followed by correlation with Spearman correlation coefficient. **(B)** MuSK IgG4 concentrations in patients with and without immunosuppressive treatments. Patients from Greece are indicated by blue symbols. Patients who remained untreated >6 months after disease onset are indicated by orange symbols. ns= non-significant. *N* = 3. Mann–Whitney test.

To this aim, we measured MuSK IgG4 concentrations from 44 patients with MuSK-MG (including the five Greek patients in the before treatment group) by ELISA. MuSK IgG4 levels correlated significantly with the MuSK IgG levels (p = 0.0011, linear regression, Spearman correlation, **Supplementary Figure 2B**) and MGFA scores (p = 0.026, linear regression, Spearman correlation, **Figure 3A**). However, no significant difference between treated and untreated patients was found (p = 0.96, Mann–Whitney test, **Figure 3B**). We hypothesized that the cause of this unexpected finding could result from the heterogeneity of the untreated patients’ cohort. We further analyzed the group and defined two subgroups: patients with MuSK-MG at onset (≤6 months after onset) and patients with MuSK-MG later in the disease course (>6 months after onset, **Supplementary Figure 3A**). At disease onset, the patients had a trend for higher MuSK IgG4 levels and more severe disease manifestations (**Supplementary Figures 3A, B**), with significantly higher MGFA compared to the scores from patients included later during disease (*p* = 0.040, Mann–Whitney test, **Supplementary Figure 3C**). Furthermore, disease severity significantly correlated with MuSK IgG4 levels in these patients (*p* = 0.022, linear regression, Pearson correlation coefficient, **Supplementary Figure 3D**).

**Figure 4:**
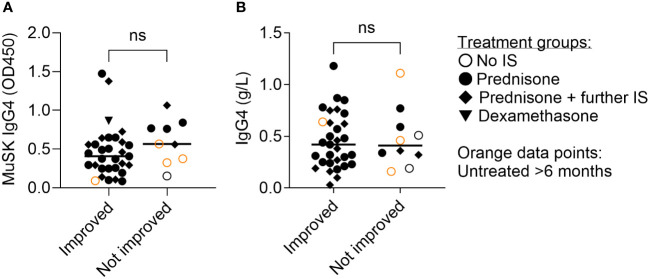
Clinical improvement was not associated with significantly reduced MuSK IgG4 **(A)** or total IgG4 concentrations **(B)**. Average disease duration: 10.3 years (range, 0–34 years, details in **Supplementary Table 1**). ns= non-significant. Mann–Whitney test.

We were uncertain whether the apparent lack of reduction in MuSK IgG4 levels in patients undergoing immune therapy stemmed from a less aggressive treatment approach or if, in fact, there was a reduction but from much higher initial antibody levels following clinical improvement. Therefore, we compared MuSK IgG4 and total IgG4 concentrations in patients with MuSK-MG from Italy, Belgium, and the Netherlands with and without clinical improvement. We did not see a significant difference in MuSK IgG4 or IgG4 levels between the groups (**Figures 4A, B**). However, we observed a trend for higher MuSK IgG4 (but not total IgG4) levels in patients without clinical improvement. The data exhibited substantial variability, and the low sample size might have obscured any underlying effect.

Linear regression models assessing the relationship between immunosuppressive therapy and MuSK IgG4 levels revealed no significant association (β = 0.017, *p* = 0.951; **Supplementary Table 1a** and **Supplementary Figure 7**). The addition of covariates sex, age, and MGFA scores did not change this relationship (**Supplementary Table 1b**).

Taken together, this implies that the variability of MuSK IgG4 levels among different patients is more pronounced than changes within individual patients. Consequently, performing longitudinal testing on individual patients might offer more valuable insights for prognosis compared to comparing sera across different patients.

### 3.3 IgG and IgG4 concentrations in patients with and without immune therapy

Next, we assessed IgG and IgG4 concentrations in patients with MuSK-MG and AChR-MG in comparison to non-disease controls.

**Figure 5:**
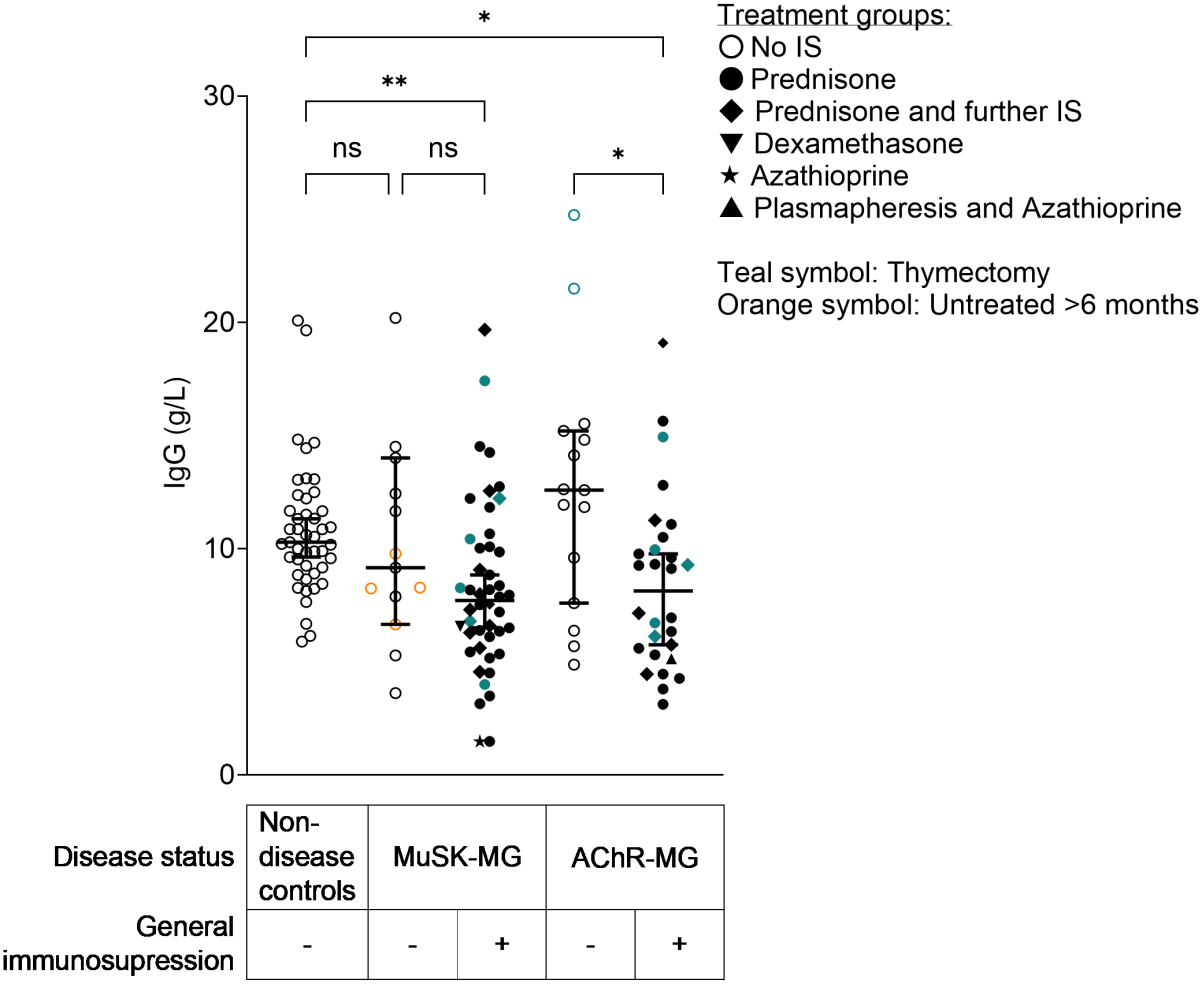
IgG concentrations in patients with MuSK-MG and AChR-MG with and without immunosuppressive treatment and non-disease controls. IgG concentrations were measured by IgG ELISA. **Non-disease control vs. MuSK-MG with immunosuppression: *p* = 0.025. *AChR-MG without vs. AChR-MG with immunosuppression: *p* = 0.036. *Non-disease control vs. AChR-MG with immunosuppression: *p* = 0.025. Kruskal–Wallis test with Dunn’s multiple comparisons test. ns= non-significant.

As expected, patients with AChR-MG receiving immunosuppressive treatment had lower IgG concentrations than untreated patients with AChR-MG (*p* = 0.036). Compared to non-disease controls, IgG concentrations were lower in patients with MuSK-MG with immunosuppression (*p* = 0.025) and patients with AChR-MG with immunosuppression (*p* = 0.025). Treated patients with MuSK-MG had lower IgG levels than untreated patients, but the difference did not reach statistical significance (**Figure 5**, Kruskal**–**Wallis test with Dunn’s multiple comparisons test). Five of the patients with MuSK underwent thymectomy prior to diagnosis of MuSK MG (patients indicated in teal symbols in **Figure 5**), and histology showed that all had a normal or atrophic thymus.

**Figure 6:**
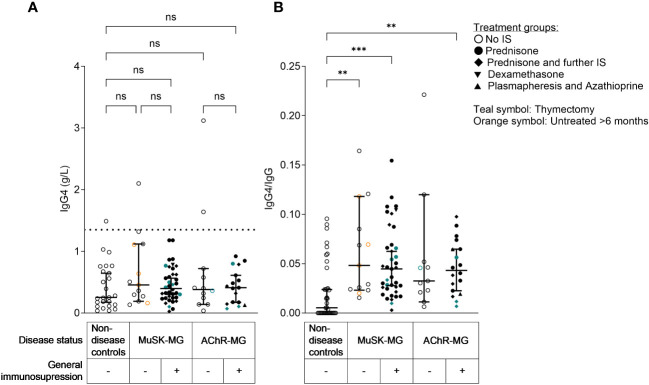
Serum IgG4 concentrations in patients with MG were, with a few exceptions, within the normal range, while relative IgG4 levels (IgG4/IgG) were enriched in patients with AChR-MG and MuSK-MG. **(A)** IgG4 serum concentrations in patients with MG and controls, Kruskal-Wallis test with Dunn’s multiple comparisons test. The dotted line indicates the cut-off for elevated IgG4 levels (defined as 1.35 g/L). **(B)** Relative IgG4 levels (IgG4/IgG), Kruskal–Wallis test with Dunn’s multiple comparisons test. **Non-disease controls vs. MuSK-MG untreated: *p* = 0.0038; ***non-disease controls vs. MuSK treated: *p* = 0.0002; **non-disease controls vs. AChR-MG treated: *p* = 0.0051. ns= non-significant.

Next, we assessed total IgG4 concentrations in sera from non-disease controls and patients with AChR-MG and MuSK-MG and observed no significant correlation between IgG4 concentrations and disease severity in patients with MuSK-MG (**Supplementary Figure 4A**) or in total IgG4 concentrations between patients with MuSK-MG and those with AChR-MG with and without immunosuppressive treatment (**Figure 6A**). Additionally, total IgG4 serum concentrations did not correlate with the MuSK IgG concentration measured by RIA (**Supplementary Figure 4C**). Total IgG4 levels were not significantly different between patients with MG and non-disease controls (**Supplementary Figure 4B**). Overall IgG4 concentrations only exceptionally exceed the threshold value of 1.35 g/L (indicated as a horizontal dotted line, **Figure 6A**), which is used to indicate elevated serum IgG4 levels in patients with IgG4-related diseases. We also observed that, intriguingly, both patients with AChR-MG and those with MuSK-MG showed an enrichment of IgG4 (**Figure 6B**, fold change of mean IgG4/IgG ratio in patients compared to non-disease controls: AChR = 2.58; AChR + IS = 2.17; MuSK = 2.91; MuSK + IS = 2.40), with no significant difference between MuSK-MG and AChR-MG. We also analyzed whether age and sex affected the IgG4 concentrations but found no correlation between age and IgG4 concentrations in patients with MuSK-MG or non-disease controls (**Supplementary Figure 5**). We observed, as expected, a trend for higher IgG4 levels in male patients in most groups (**Supplementary Figure 6**), though interestingly the three patients with MG (two with AChR-MG and one with MuSK-MG) with elevated IgG4 were women (**Supplementary Figure 6B**).

Furthermore, when linear regression models were used, no statistically significant relationship was identified between immunosuppressive therapy and total IgG4 levels (β = 0.023, *p* = 0.897; **Supplementary Table 2a** and **Supplementary Figure 8**). The association remained statistically insignificant when sex, age, and MGFA were introduced as covariates (**Supplementary Table 2b**). Furthermore, linear regression models assessing the relationship between total IgG4 levels and disease status compared to non-disease controls showed no significant association with either AChR-MG (β = 0.282, *p* = 0.256) or MuSK-MG (β = 0.331, *p* = 0.140; **Supplementary Table 3a** and **Supplementary Figure 9**). Moreover, the addition of the covariates age, sex, and immunosuppressant therapy did not change this relationship (**Supplementary Table 3b**).

### 3.4 Clinical severity over long disease duration

**Figure 7:**
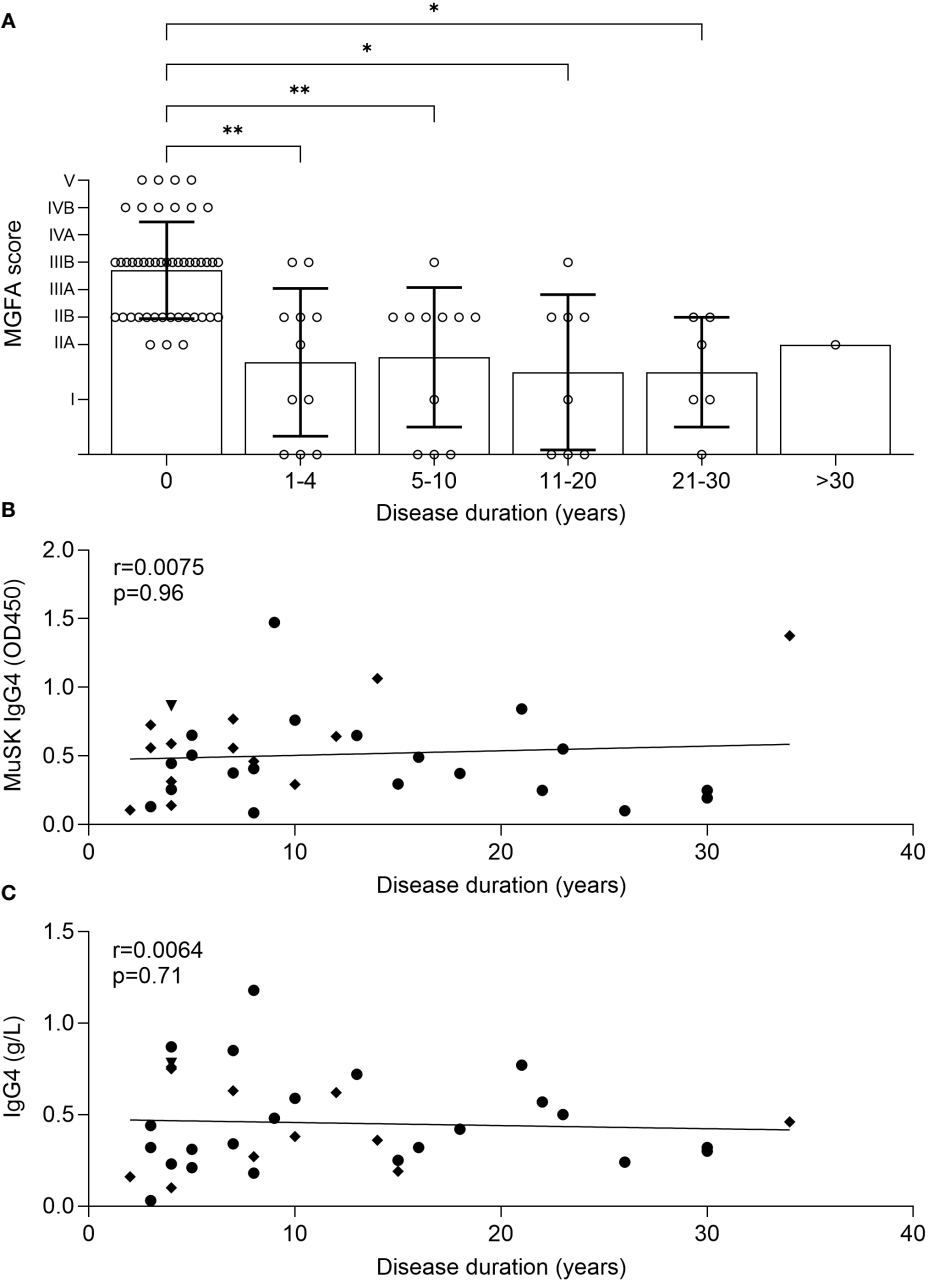
Analysis of MGFA scores showed a significant clinical improvement in the first 4 years of disease, but no further improvement afterwards **(A)**, Kruskal–Wallis test (adjusted *p*-values: 0 vs. 1–4: *p* = 0.005 (**); 0 vs. 5–10: *p* = 0.0094(**); 0 vs. 11–20: *p* = 0.013 (*); 0 vs. 21–30: *p* = 0.01 (*). Clinical remission was indicated by an MGFA score of “0” to allow inclusion in the dataset. Simple linear regression with Spearman correlation. No significant reduction of MuSK IgG4 and IgG4 levels over time **(B, C)**.

The Pisa cohort included patients with follow-up times of over 30 years. We therefore wanted to investigate how disease duration affected the clinical severity, MuSK IgG4, and total IgG4 concentrations. We observed that there was a significant reduction of MGFA scores in patients in the first 1–4 years after onset (**Figure 7A**), which remained stable with no further improvement afterwards. MuSK IgG4 (**Figure 7B**) and IgG4 concentrations (**Figure 7C**) also did not significantly change over time.

## 4 Discussion

MuSK-MG is caused by pathogenic IgG4 autoantibodies (6, 9), and MuSK antibody levels correlate with disease severity (24). Patients with MuSK-MG are often treated with general immunosuppression (mostly with prednisone and azathioprine), but the total IgG4 and MuSK IgG4 levels in patients with general immunosuppression had not yet been studied.

In this cross-sectional study, we investigated disease severity, MuSK IgG and IgG4 levels, as well as total IgG and IgG4 concentrations in patients at disease onset and during follow-up with and without immunosuppression. We made five main observations: (1) Patients with MuSK-MG showed a robust clinical improvement and reduction of MuSK IgG after therapy (average treatment time: 11.5 years, range: 0–34 years), but only 14/52 patients (26.9%) were in remission at the time of the analysis; (2) MuSK IgG4 concentrations, but not total IgG4 concentrations, correlated with clinical severity and total MuSK IgG levels; (3) MuSK IgG4 concentrations were reduced after immunosuppression in four out of five individuals with before–after data, but data from non-linked patients showed that inter-patient variability is greater than the effect in individual patients; (4) total IgG4 levels were within the normal range in all patients with MG, with few exceptions from all groups, with a relative enrichment of IgG4/IgG in both patients with AChR-MG and those with MuSK-MG; and (5) patients improved within the first 4 years after disease onset and remained stable with no further clinical improvement or MuSK IgG4 reduction.

Based on our observations, we hypothesize that (1) patients with MuSK-MG improved clinically with general immunosuppression especially during the first 4 years of treatment, but the majority did not reach clinical remission and therefore may require treatment alternatives; (2) longitudinal testing of MuSK-IgG4 in individual patients may have greater merit than comparing single-time-point measurements across patients; and (3) further studies with larger cohorts are necessary to assess serum IgG4 concentrations in patients with MuSK-MG and enrichment of total IgG4/IgG since these were not significantly different between patients with AChR-MG and those with MuSK-MG in our study.

### 4.1 Clinical response and MuSK IgG4 concentrations after immunosuppression

While it has been described that treatment with rituximab has greater clinical benefit for patients with MuSK-MG than general immunosuppression (15, 16), studies investigating the effect of general immunosuppression, particularly on MuSK IgG4 levels, are lacking. We observed a significant clinical improvement after general immunosuppression, indicated as reduction in the MGFA score, in our patient cohort. Nevertheless, clinical remission was only achieved in 12/42 treated patients with available before–after treatment data (10/36 from Italy, 0/1 from Belgium, and 2/5 from Greece), and clinical improvement was mostly observed within the first 4 years of disease duration, suggesting that a substantial fraction of patients might benefit from additional treatment.

We expected to see lower MuSK IgG4 levels in patients receiving treatment compared to untreated patients because (1) IgG4 is the predominant subclass of MuSK autoantibodies that directly cause the disease (6–9); (2) in line with previous publications (6, 24), MuSK IgG correlated with disease severity; (3) and so did MuSK IgG4 in our study; and finally (4) treatment led to clinical improvement and reduction of MuSK IgG. Upon analyzing four patients with MuSK-MG from Greece before and after receiving immunosuppression, we noted a tendency towards decreased MuSK IgG4 levels alongside clinical improvement. However, this trend could not be replicated in a more extensive cohort with cross-sectional data. Subsequent examination of untreated patients with MuSK-MG unveiled substantial heterogeneity within this group. Coupled with a notable inter-patient variability in treatment responses, these factors have influenced the data, underscoring the notion that longitudinally testing MuSK IgG4 in individual patients might offer greater clinical insights than analyzing a single measurement per patient. This is in line with a recent study investigating the effects of rituximab on MuSK IgG4 concentrations with longitudinal sampling, where a substantial inter-patient variability in MuSK IgG4 concentrations was overcome with normalization to individual patient baseline levels (22). The study also showed a correlation between MuSK IgG4 levels and clinical response to rituximab, which, together with our findings in the Greek patients, suggests that MuSK IgG4 is a suitable prognostic biomarker when used for intra-patient analysis.

### 4.2 Enrichment of IgG4 is not associated with IgG4 autoimmunity

MuSK-MG is an IgG4-AID (11, 25); therefore, the study of IgG4 serum levels may give valuable insights into the immunopathogenesis and etiology (13). An open question in the field is why the autoantibodies in these diseases are mainly of the IgG4 subclass, and one hypothesis is that an immune dysregulation may lead to a skewed IgG subclass profile and increased production of IgG4 and total IgG (13). No indication for increased IgG production could be found in our study, as there was no significant difference in total IgG between non-disease controls and untreated patients with MuSK-MG, which is in line with previous studies (26, 27).

In contrast, a recent study showed an inverse correlation between AChR autoantibody levels and clinical improvement in patients with AChR-MG over time (28). The authors highlight the potential of measuring AChR autoantibody levels as an objective measurement to evaluate treatment efficacy and allow timely changes in the immunosuppressive treatment selected to prevent unnecessary delays in individual patients. It is also essential, for the accurate quantification of the autoantibodies, to include a serial dilution of the patient’s serum or plasma. The lack of these or other controls makes it difficult to draw a conclusion on the impact of IgG and antigen-specific autoantibody levels in the pathology across the literature.

We next studied whether IgG4 levels were increased in patients with MuSK-MG. In our study, four samples were above the threshold of 1.35 g/L: 1/52 (1.9%) patients with MuSK-MG [1/13 (7.7%) untreated patients with MuSK-MG], 2/43 (4.6%) patients with AChR-MG [2/15 (13.3%) untreated], and 1/45 (2.22%) non-disease controls. This is in contrast to a recent study by Vergoossen et al. where 22% of 28 untreated patients with MuSK-MG showed above-threshold IgG4 concentrations (26). Both studies had low numbers of patients due to the rarity of disease, and this may account for the variation. Furthermore, we also did not observe a correlation between total IgG4 and disease severity, while MuSK IgG and disease severity correlated. We then explored the possibility of a relative enrichment of IgG4, combining reduced total IgG levels with stable/higher total IgG4, and observed a significant IgG4/IgG enrichment, which is in line with one previous study (26), but in contrast to another study with no enrichment (27). It is tempting to speculate that differences in IgG1–IgG3 versus IgG4 expression may be influenced by a different inflammation status in IgG4 autoimmunity in contrast to classic IgG1 autoimmunity. In line with this hypothesis, the Vergoossen study observed a relative IgG4 enrichment specific for MuSK-MG that was not observed in AChR-MG. Nonetheless, we and others found elevated serum IgG4 levels and enrichment of IgG4/IgG in patients with AChR-MG (29). We observed a trend for a sub-threshold (<1.35 g/L) increase of total IgG4 levels in both patients with AChR-MG and those with MuSK-MG with and without immunosuppression, and an enrichment of IgG4/IgG in both patients with AChR-MG and those with MuSK-MG, which is in line with the study of Liu and colleagues (29). However, it is possible with the data at hand to agree with the main conclusions of the abovementioned studies (26, 30), that a significant elevation of serum IgG4, as observed as in IgG4-related diseases, is not observed in MuSK-MG.

What may have caused the discrepant results? Different geographic regions, and thus different genetic backgrounds, as well as technical differences in the determination of IgG and MuSK-specific IgG concentrations may also play a role. Immunosuppression may have affected IgG4 levels, but we also included untreated patients and covariate analysis did not show an effect of treatment on IgG4 levels. The discrepancy in data interpretation is also likely to derive from the data transformation that was applied. Since our data were not normally distributed, we log-transformed the data before regression analyses. When the analyses were performed without the log transformation, the results resembled the Vergoossen study more (26), indicating that differences in the statistical approach may have caused the differences. The enrichment of serum IgG4 in AChR-MG and MuSK-MG is interesting, as mild elevation of IgG4 could be a characteristic of MG in general, as it is also in a range of disorders from cancer to allergy and rheumatoid arthritis (31–35). However, we observed that IgG4 concentrations did not correlate with MuSK IgG concentrations, disease severity, or disease duration in patients with MuSK-MG. Therefore, we conclude that aberrant IgG4 production is unlikely to be the main driver of immunopathogenesis and that MuSK IgG4 only comprises a minor fraction of the total IgG4 antibody repertoire. Furthermore, the presence of individuals with elevated IgG4 (>1.35 g/L) may be common across populations, as IgG4 is known to be highly variable in healthy individuals, ranging between 0.01 and 1.4 mg/mL, dependent on age, sex, and ethnicity, and may seasonally change, e.g., during allergy/infection seasons (36–39). Interestingly, a recent study showed that tobacco smoking is associated in a dose-dependent manner with elevated serum IgG4 levels (40). Smoking after disease onset is associated with an increase in disease severity and the progression towards generalized muscle weakness (41–43), but to date, the effect of smoking on IgG4 in MuSK-MG has not been investigated, and it is possible that tobacco use is a confounding factor for the analysis of serum IgG4.

### 4.3 Limitations

A key limitation of our study is the fluctuating nature of MuSK-MG, with clinical severity changing over time. Moreover, MG has a low disease prevalence, and therefore, sample numbers were limited. This is a limitation of the study, and findings require to be validated with larger patient numbers in the future. A further limitation of the study is that the clinical severity was assessed only via MGFA scores and not by a more quantitative score such as QMG. Furthermore, owing to technical reasons, MuSK IgG levels assessed at the local diagnostic centers in Pisa and the Neuroimmunology group in Oxford differed from each other (**Supplementary Tables**). The RIA measurements from Oxford of the patients from Pisa correlated well with the results from our ELISA, suggesting a higher accuracy of the RIA. Therefore, quantitative analyses used the measurements by the RIA measurements from Oxford. The local measurements from Pisa were only used in **Figure 1** to investigate the overall change in MuSK IgG levels over time (**Figure 1B**).

## 5 Conclusion

General immunosuppression had a clinical benefit for patients with MuSK-MG during the first 4 years of disease duration, but only 13/44 patients with general immunosuppression or 14/52 patients with MuSK-MG in total reached clinical remission, suggesting that other or more intense treatments may be necessary for a substantial fraction of patients with MuSK-MG to reach remission. The immunosuppressive treatment also led to a reduction of MuSK IgG4 in four out of five individual patients, but could not be observed across patients, probably due to inter-patient variability. Serum IgG4 was within the normal range, but relatively enriched (IgG4/IgG) in both patients with AChR-MG and those with MuSK-MG. Owing to the low sample size, further studies with larger cohorts and longitudinal sampling are required to validate these findings.

## Data availability statement

The original contributions presented in the study are included in the article/**Supplementary Material**. Further inquiries can be directed to the corresponding authors.

## Ethics statement

The studies involving humans were approved by Institutional Review Boards of the Medical University of Vienna, Austria (EK 1442/2017). The studies were conducted in accordance with the local legislation and institutional requirements. Written informed consent for participation in this study was provided by the participants’ legal guardians/next of kin.

## Author contributions

IK: Conceptualization, Data curation, Formal analysis, Funding acquisition, Investigation, Methodology, Resources, Supervision, Validation, Visualization, Writing – original draft, Writing – review & editing. MMD: Data curation, Investigation, Methodology, Writing – review & editing. SZ: Data curation, Investigation, Methodology, Writing – review & editing. SDH: Data curation, Investigation, Methodology, Writing – review & editing. SH: Writing – review & editing. DVK: Formal analysis, Validation, Visualization, Writing – original draft, Writing – review & editing. JD: Data curation, Supervision, Writing – review & editing. ADR: Writing – review & editing, Resources. MM: Resources, Writing – review & editing. MG: Resources, Writing – review & editing. PM: Writing – review & editing. PVD: Writing – review & editing, Resources. AF: Writing – review & editing, Data curation, Supervision. TP: Supervision, Writing – review & editing. MDB: Writing – review & editing, Supervision. KL: Resources, Writing – review & editing. VZ: Writing – review & editing, Resources. ST: Resources, Writing – review & editing. RR: Resources, Writing – review & editing. ML: Resources, Writing – review & editing, Conceptualization, Data curation, Formal analysis, Methodology, Supervision, Validation, Visualization, Writing – original draft. PMM: Conceptualization, Supervision, Writing – original draft, Writing – review & editing, Investigation, Project administration.
