# Supplementary material for: A retrospective multicenter study on clinical and serological parameters in patients with MuSK myasthenia gravis with and without general immunosuppression
Source: Front Immunol. 2024 Apr 23;15:1325171. doi: 10.3389/fimmu.2024.1325171 (PMC11074957; doi:10.3389/fimmu.2024.1325171)
Supplement: Supplementary file 1 [file DataSheet_1.pdf]

Supplementary Material

A multicenter retrospective study on clinical and serological response to general immunosuppression in MuSK myasthenia gravis patients

Inga Koneczny[1,2], Marina Mané-Damas[1#], Shenghua Zong[1#], Sander De Haas[1#], Saif Huda[3], Daan van Kruining[1], Jan Damoiseaux[1], Anna de Rosa[4], Michelangelo Maestri[4], Melania Guida[4], Peter Molenaar[1], Philipp van Damme[6,7], Andreas Fichtenbaum[8,2], Thomas Perkmann[8], Marc De Baets[1], Konstantinos Lazaridis[5], Vasiliki Zouvelou[10], Socrates Tzartos[5,9], Roberta Ricciardi[4], Mario Losen[1#], Pilar Martinez-Martinez[1#]

*** Correspondence:**
Pilar Martinez-Martinez and Mario Losen, Department of Psychiatry and Neuropsychology, School for Mental Health and Neuroscience, Maastricht University, Universiteitssingel 50, 6229 ER Maastricht, the Netherlands. Email: p.martinez@maastrichtuniversity.nl; m.losen@maastrichtuniversity.nl

1 Supplementary Data

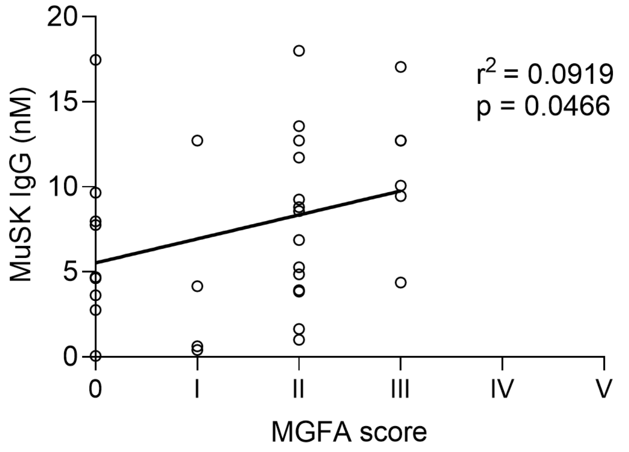

Supplementary figure 1: MuSK IgG and MGFA score significant correlation. Available serum samples were titrated by RIA in Oxford and the MuSK IgG titer correlated to the MGFA score. Linear regression followed by correlation with Pearson correlation coefficient.

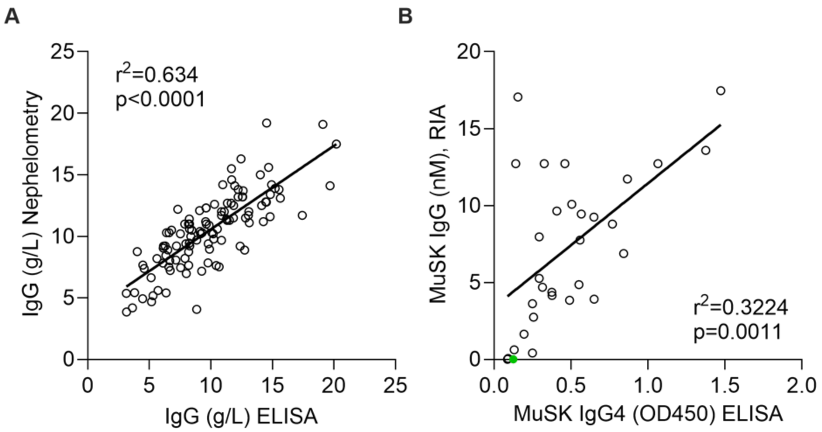

Supplementary figure 2: Quantitative correlation across assays. **(A)** Correlation nephelometry/ELISA. **(B)** MuSK IgG assessed by RIA (Oxford) were plotted against MuSK IgG4 assessed by ELISA. N=3. Black= MuSK MG patients. Green = non-disease control. Linear regression followed by correlation with Pearson correlation coefficient.

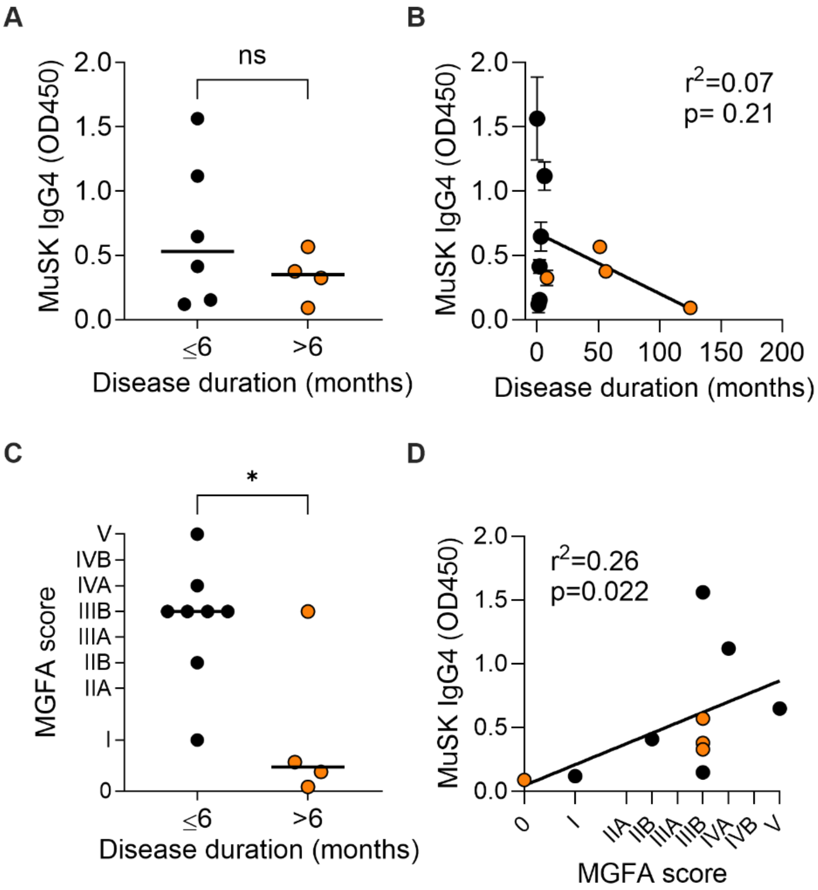

Supplementary figure 3: Untreated MuSK-MG patients consist of two distinct subgroups with more severe disease and higher MuSK IgG4 at onset and mild disease and lower MuSK IgG4 during disease course. **(A, B)** Trend for reduced MuSK IgG4 levels over time in untreated patients. **(A)** MuSK IgG4 levels at disease onset (≤6 months after onset, black symbols) and later disease course (>6 months after onset, orange symbols), Mann-Whitney test, p= 0.352. **(B)** Linear regression followed by correlation with Spearman correlation coefficient. **(C)** MGFA scores at disease onset (≤6 months after onset, black symbols) and later disease course (>6 months after onset, orange symbols), Mann-Whitney test, p= 0.040; one sample was excluded as no data for exact disease duration was available. **(D)** MuSK IgG4 levels correlated with disease severity in untreated MuSK-MG patients, linear regression followed by correlation with Spearman correlation coefficient.

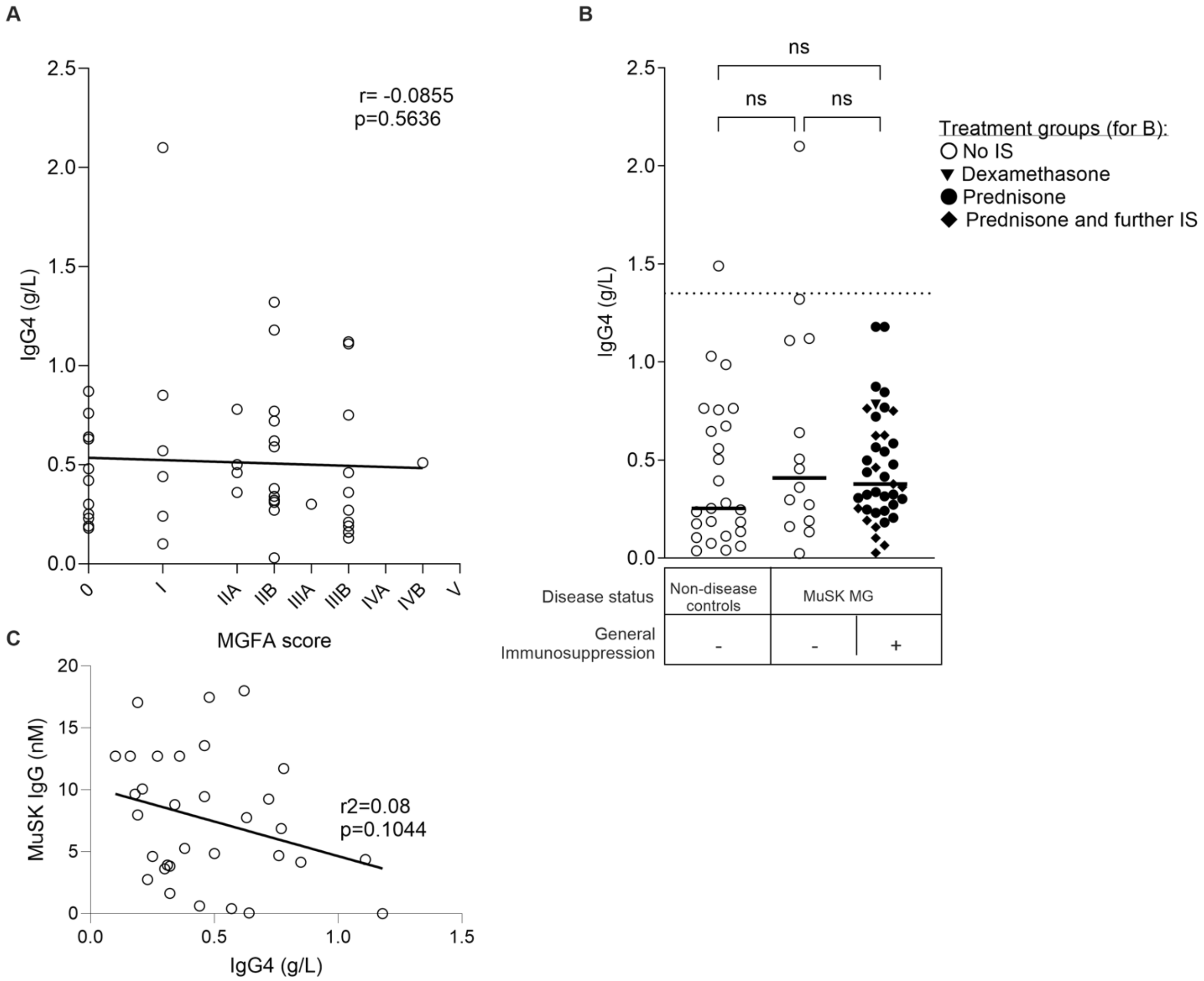

Supplementary figure 4: Serum IgG4 concentrations in MuSK-MG patients did not correlate with clinical severity or treatment status and were within the normal range. **(A)** No correlation of IgG4 with clinical severity. Linear regression followed by correlation with Spearman correlation coefficient. **(B)** A trend for total IgG4 upregulation in MuSK-MG patients in comparison to non-disease controls can be observed, but we see no difference between treated and untreated patients. Kruskal-Wallis test with Dunn's post-test. **(C)** No correlation between total IgG4 concentrations and MuSK IgG serum levels. IgG4 (g/L) was measured by nephelometry, MuSK IgG (nM) by RIA. Linear regression with Spearman correlation.

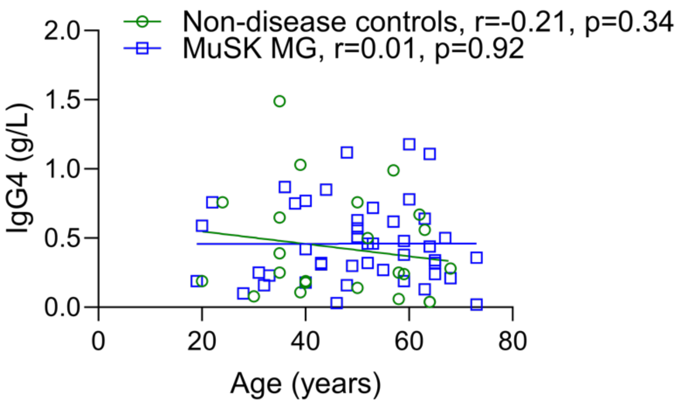

Supplementary figure 5: No correlation between IgG4 concentrations and age. Green: non-disease controls from the Netherlands, blue: MuSK-MG patients from Pisa. Linear regression followed by correlation with Spearman correlation coefficient.

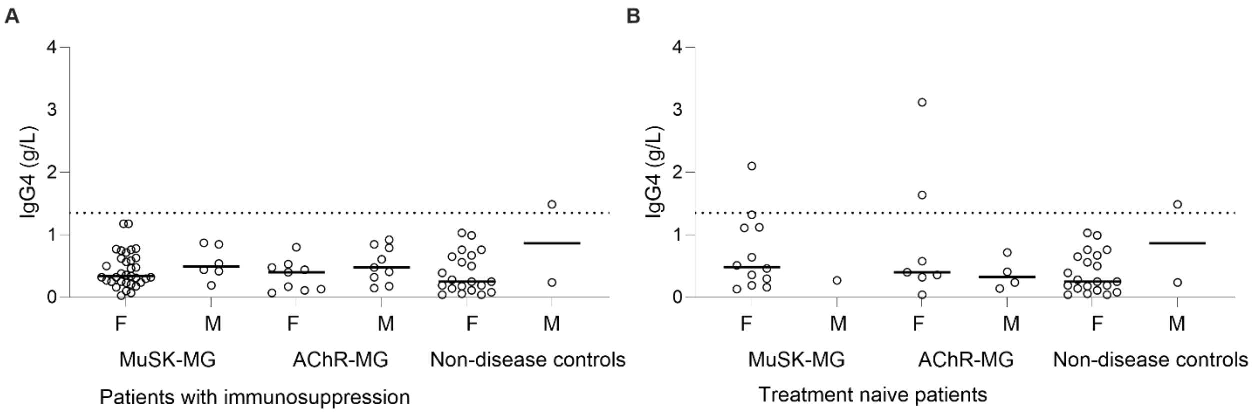

Supplementary figure 6: Serum IgG4 concentrations in female and male patients with **(A)** and without **(B)** immunosuppressive treatment. Kruskal Wallis test with Dunn's post test.

Covariance analysis

Linear regression models assessing the relationship between immunosuppressive therapy and MuSK IgG4 levels revealed no significant association (β = 0.017, p = 0.951; **Supplementary table 1a, Supplementary Fig. 7**). The addition of covariates sex, age, and MGFA scores did not change this relationship (**Supplementary table 1b**).

Supplementary table 1. Multivariable-adjusted associations of immunosuppressive therapy and MuSK IgG4 levels.

a.

Coefficient	Estimate	Std.Error	t-value	P-value
(Intercept)	***-0.983***	***0.240***	***-4.098***	***0.000***
IS therapy	0.017	0.268	0.062	0.951

b.

Coefficient	Estimate	Std.Error	t-value	P-value
(Intercept)	***-1.594***	***0.468***	***-3.408***	***0.002***
IS therapy	0.248	0.291	0.854	0.398
age	0.003	0.008	0.321	0.750
sexM	0.148	0.356	0.417	0.679
MGFA	*0.183*	*0.101*	*1.811*	*0.078*

a) Model 1: Crude (no adjustments)
b) Model 2: Model 1 + age, sex, and MGFA scores

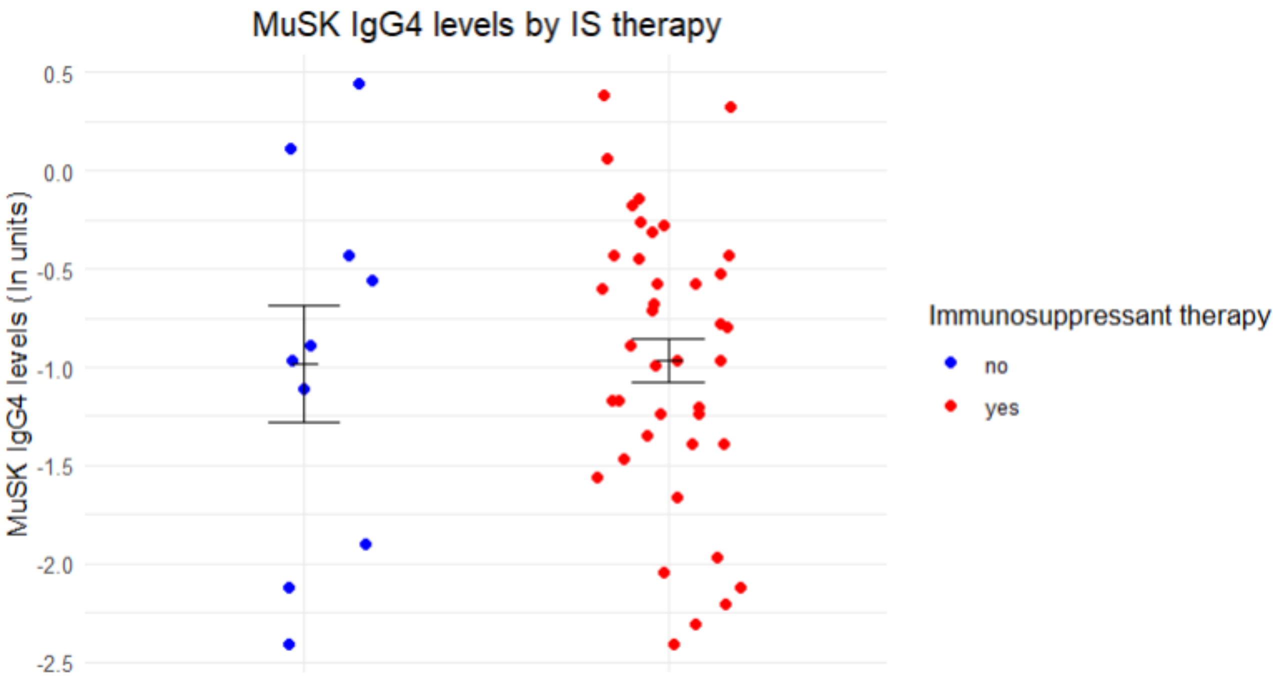

Supplementary figure 7. MuSK IgG4 level distributions between patients receiving immunosuppressant (IS) therapy and those without treatment. MuSK IgG4 levels in patients receiving IS therapy (red) do not significantly differ from those receiving no treatment (blue) ($p = 0.951$).

Additionally, no statistically significant relationship was identified between immunosuppressive therapy and total IgG4 levels ($\beta = 0.023$, $p = 0.897$; **Supplementary table 2a, Supplementary figure 8**). The association remained statistically insignificant when sex, age, and MGFA were introduced as covariates (**Supplementary table 2b**).

Supplementary table 2. Multivariable-adjusted associations of immunosuppressive therapy and total IgG4 levels.

a.

Coefficient	Estimate	Std.Error	t-value	P-value
(Intercept)	***-1.050***	***0.130***	***-8.030***	***0.000***
IS therapy	0.023	0.176	0.130	0.897

b.

Coefficient	Estimate	Std.Error	t-value	P-value
(Intercept)	-0.617	0.474	-1.300	0.201
IS therapy	-0.275	0.283	-0.972	0.337
age	0.001	0.008	0.114	0.910
sexM	0.052	0.361	0.143	0.887
MGFA	-0.089	0.100	-0.890	0.379

a) Model 1: Crude (no adjustments)

b) Model 2: Model 1 + age, sex, and MGFA scores

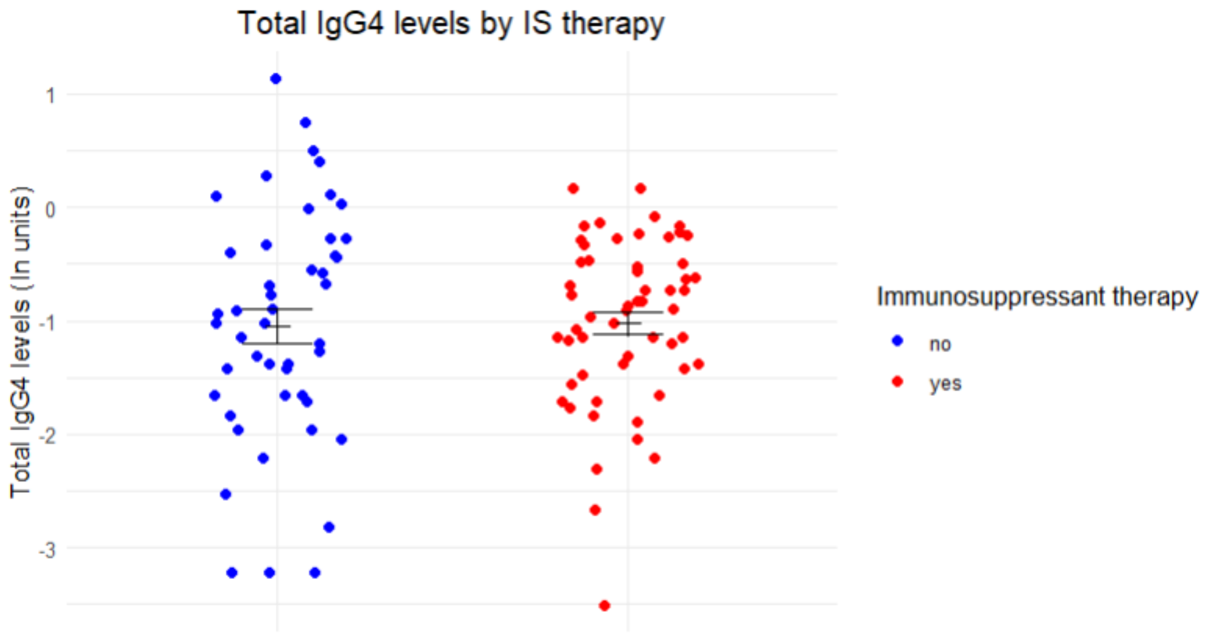

Supplementary figure 8. Total IgG4 level distributions between patients receiving immunosuppressant (IS) therapy and those without treatment. Total IgG4 levels in patients receiving IS therapy (red) do not significantly differ from those receiving no treatment (blue) ($p = 0.897$).

Furthermore, linear regression models assessing the relationship between total IgG4 levels and disease status compared to healthy controls showed no significant association with either AChR MG ($\beta = 0.282$, $p = 0.256$) or MuSK MG ($\beta = 0.331$, $p = 0.140$; **Supplementary table 3a, Supplementary figure 9**). Moreover, the addition of the covariates age, sex, and immunosuppressant therapy did not change this relationship (**Supplementary table 3b**).

Supplementary table 3. Multivariable-adjusted associations of total IgG4 levels and disease status (AChR-MG or MuSK-MG).

a.

**Coefficient**	**Estimate**	**Std.Error**	**t-value**	**P-value**
(Intercept)	***-1.290***	***0.185***	***-6.980***	***0.000***
AChR MG status	0.282	0.247	1.140	0.256
MuSK MG status	0.331	0.222	1.490	0.140

b.

**Coefficient**	**Estimate**	**Std.Error**	**t-value**	**P-value**
(Intercept)	***-0.975***	***0.324***	***-3.014***	***0.003***
AChR MG status	0.384	0.295	1.299	0.197
MuSK MG status	*0.493*	*0.279*	*1.769*	*0.080*
Age	-0.007	0.006	-1.268	0.208
Sex	0.352	0.237	1.486	0.141
IS therapy	-0.207	0.222	-0.933	0.353

a) Model 1: Crude (no adjustments)
b) Model 2: Model 1 + age, sex, and immunosuppressant (IS) therapy

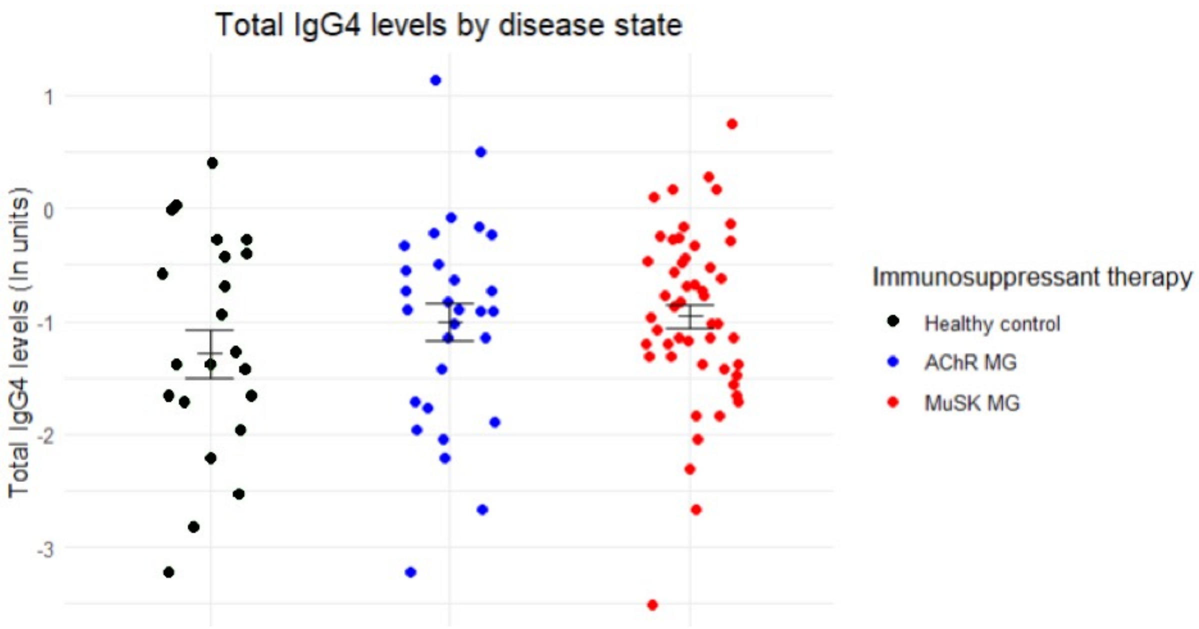

Supplementary figure 9. Total IgG4 level distributions between disease states. No significant differences in total IgG4 levels were observed between healthy controls (black) and AChR-MG (blue) or MuSK-MG (red) patients.
